# Roux en Y gastric bypass and iterative intussusception at the jejuno-jejunal anastomosis: Conversion into one anastomosis gastric bypass (with video) - A case report

**DOI:** 10.1016/j.ijscr.2024.109244

**Published:** 2024-01-10

**Authors:** Arnaud Liagre, Francesco Martini, Tarek Debs, Sara Claudia Barone, Niccolo Petrucciani

**Affiliations:** a Clinique Des Cedres, Bariatric Surgery Unit, Ramsay Générale de Santé, Cornebarrieu, France; b Institut Arnault Tzanck, 116 Rue du Commandant Gaston Cahuzac, 06700 Saint-Laurent-du-Var, France; c Department of Medical and Surgical Sciences and Translational Medicine, Faculty of Medicine and Psychology, St Andrea Hospital, Sapienza University, Rome, Italy

**Keywords:** Roux en Y gastric bypass, One anastomosis gastric bypass, Intussusception, Laparoscopic

## Abstract

**Introduction:**

The aim of this article is to describe a rare complication of Roux en Y gastric bypass (RYGB): recurrent intestinal intussusception of the biliary limb, and an original treatment: the removal of the jejuno-jejunal anastomosis with conversion into “short limb” one anastomosis gastric bypass (OAGB).

**Presentation of case:**

A 25-year-old patient underwent RYGB fashioned with a 50 cm-length biliary loop and a 150 cm-length alimentary loop. She was hospitalized other 3 times in the following months for episodes of acute abdominal pain and excessive weight loss, with CT scans showing intussusception at the jejuno-jejunal anastomosis. Conversion from RYGB to OAGB with “short biliary limb” was performed. The patient at 60-month follow-up has no bile reflux and regained weight.

**Discussion:**

Small bowel intussusception is a rare complication that can occur following Roux-en-Y gastric bypass (RYGB) surgery, leading to symptoms like acute or chronic abdominal pain. Treatment options reported in medical literature include resection and re-fashioning of the jejuno-jejunal anastomosis, simple reduction (with a risk of recurrence), and imbrication/plication of the jejuno-jejunal anastomosis. Given the rarity of this complication, there are no standardized recommendations, and the best treatment should be determined on a case-by-case basis, taking into consideration the patient's unique circumstances and the medical team's expertise.

**Conclusion:**

Intestinal intussusception at the jejuno-jejunal anastomosis responsible for chronic abdominal pain is a rare complication after RYGB. One of the possible treatments is conversion into OAGB.

## Highlights

• Chronic intussusception at the jejuno-jejunal anastomosis is a rare complication after RYGB
• The diagnosis is based on patients' symptoms and CT scan findings
• Conversion from RYGB to OAGB is a possible effective treatment

## 1 Introduction

The Roux Y gastric bypass (RYGB) is the reference technique for obesity's surgical treatment (1). The re-establishment of bowel continuity is usually achieved by an anisoperistaltic anastomosis between the biliary and alimentary limbs. Jejunal intussusception following RYGB represents a rare complication after RYGB with a pooled incidence of 0.64 % recently reported by a meta-analysis including 191 patients in 74 studies (2). Considering a reported rate of complications of 4.4 % and reoperations of 2.8 % (3), compared to other causes of reoperation, intussusception is rare. Resection of the affected segment is reported in 34 % of the patients with intussusception and recurrence occurs in 22 % of cases (2). Resection and reconstruction of the jejunojejunostomy appears to be associated with the lowest risk of recurrence.

The aim of this case report is to describe a rare complication of RYGB: recurrent intestinal intussusception of the biliary limb and the removal of the jejuno-jejunal anastomosis with conversion into “short limb” one anastomosis gastric bypass (OAGB) (4,5). This case was reported in accordance with the SCARE criteria (6).

## 2 Presentation of case

A 25-year-old female patient underwent RYGB in 2008, fashioned with a 50 cm-length biliary loop and a 150 cm-length alimentary loop. The gastro-jejunal anastomosis was a hand-sewn termino-lateral anastomosis, whereas the jejuno-jejunal anastomosis was stapled and latero-lateral. The mesenteric defects remained not closed.

Her initial weight was 123 kg, with body mass index (BMI) of 41 kg/m2. The patient underwent cholecystectomy 6 months after RYGB for a biliary colic. The mesenteric defects were closed 9 months after surgery for an episode of left hypochondrium pain suspect for internal hernia. She was rehospitalized 10 months after RYGB for left hypochondrium pain and jejuno-jejunal intussusception of the biliary loop at computed tomography (CT) scan. An exploratory laparoscopy was performed and did not retrieve any anomalies. The mesenteric defects were still well closed.

She was rehospitalized at 84 months for intense postprandial iterative abdominal pain, excessive weight loss (53 kg, BMI 18) secondary to chronic abdominal pain and intestinal intussusception at the same location. Considering the intensity of the pain, a new exploratory laparoscopy was performed, which did not retrieve any anomalies. During the hospitalization, which lasted 10 days, the abdominal pain did not disappear but had only a minor improvement. The patient was examined again 4 months after the last hospitalization, for recrudescence of the pain. A CT scan detected a jejuno-jejunal intussusception at the jejuno-jejunal anastomosis. The diagnosis of aniso-peristaltic intestinal intussusception at the jejuno-jejunal anastomosis was made.

Considering the chronic abdominal pain causing excessive weight loss and the radiological findings, indication for revisional surgery was retained. A conversion from RYGB to OAGB with “short biliary limb” was performed. As shown in the Video, the jejuno-jejunal anastomosis was sectioned using a 60 mm stapler with vascular load and a semi-mechanical latero-lateral anastomosis between the biliary limb and the alimentary limb was performed 5 cm below the gastrojejunal anastomosis. The length of the biliary limb was not changed. The pain disappeared immediately as well as the biliary limb intussusception on the CT scan. The patient at 60-month follow-up after OAGB has no bile reflux, has regained weight (68 kg, BMI 23) and her alimentary quality of life has dramatically improved.

## 3 Discussion

Small bowel intussusception is a rare complication after RYGB and can cause acute or chronic abdominal pain months or years after the bypass (1,4,5). Treatment options reported in the literature include resection and re-fashioning of the jejuno-jejunal anastomosis (1), simple reduction, associated with the higher risk of recurrence and imbrication/plication of the jejuno-jejunal anastomosis (5). The etiology is poorly understood, and the role of the staple or suture line as a lead point or motility disorders have been advocated. In patients with milder symptoms and laboratory and radiological exams not showing signs of small bowel ischemia, non-operative management can be attempted. In case of severe pain, tenderness at examination, suspect of bowel ischemia or signs of persistent abdominal obstruction, laparoscopic exploration is recommended. The management of transient intussusception is controversial. Currently, there is no consensus as to which treatment is optimal for JI occurring after RYGB surgery (7). In the literature, the options for operative management of JI include reduction alone, reduction with enteropexy, and resection of the JJ with reconstruction of the anastomosis. In the largest series, reduction and enteropexy was the most common approach, followed by reduction only and by small bowel resection (7). Overall pooled recurrence of 22 % was reported (2), and reduction alone is associated with the higher recurrence rate (1,7). In the present article, we propose an original treatment for recurrent intussusception, conversion into one anastomosis gastric bypass (OAGB). OAGB is an established bariatric surgical procedure with results that are not inferior to those of RYGB in term of weigh loss and lower long-term morbidity (8,9). Eliminating the jejuno-jejunal anastomosis as in OAGB, the possibility of intussusception which occurs at the jejuno-jejunal anastomosis is also eliminated. Therefore it is potentially a simple and valuable treatment, especially in cases experiences several recurrences, especially in patients who already had refashioning of the anastomosis. In the present patient, we report the conversion into “short limb” one anastomosis gastric bypass, useful also for the associated excessive weight loss. We believe that considering the rarity of this complication, there are no precise recommendations and no guidelines have been published, and the best treatment must be decided on a case-by-case basis.

## 4 Conclusion

Intestinal intussusception at the jejuno-jejunal anastomosis responsible for chronic abdominal pain is a rare complication after RYGB. One of the possible treatments may be the removal of the jejuno-jejunal anastomosis by conversion into OAGB.

The following is the supplementary data related to this article.

Video Video

## Consent

Written informed consent was obtained from the patient for publication on this case report and accompanying images. A copy of the written consent is available for review by the Editor-in-Chief of this journal of request.

## Ethical approval

As no experimental treatment was performed, this study is exempt from ethical approval in our institution (La Sapienza University of Rome). The patient was treated according to the standard of care of our Hospital. Patient approval has been given.

## Funding

No sources of funding.

## Author contribution

Niccolò Petrucciani; Sara Claudia Barone; Arnaud Liagre; Francesco Martini; Tarek Debs; − Supervision, Data curation, Writing, Review and Editing.

Arnaud Liagre; Francesco Martini; Tarek Debs; Sara Claudia Barone; Niccolò Petrucciani;– Conceptualization, Data curation, Writing- Original draft.

Niccolò Petrucciani – Supervision, Data curation, Writing, Review and Editing.

All – approval of final manuscript.

## Guarantor

Niccolò Petrucciani.

## Declaration of competing interest

This research did not receive any specific grant from funding agencies in the public, commercial, or not-for-profit sectors. The authors have no competing interests to declare.

## References

[1] Latif J., Krivan S., Awan A. (2021). Laparoscopic management of Jejuno-jejunal intussusception post laparoscopic Roux-en-Y Gastric Bypass in a pregnant patient. Obes. Surg..

[2] Oor J.E., Goense L., Wiezer M.J., Derksen W.J.M. (2021 May). Incidence and treatment of intussusception following roux-en-Y gastric bypass: a systematic review and meta-analysis. Surg. Obes. Relat. Dis..

[3] Wiggins T., Pournaras D.J., Priestman E., Osborne A., Titcomb D.R., Finlay I. (2021 Jun). Effect of preoperative weight loss and baseline comorbidity on short-term complications and reoperations after laparoscopic roux-en-Y gastric bypass in 2,067 patients. Obes. Surg..

[4] Guarner P., Ibarzabal A., Balibrea J.M., Lacy A.M. (2020 May). Biliopancreatic limb obstruction due to jejuno-jejunal intussusception in a patient with previous Roux-en-Y gastric bypass. Cir. Esp. (Engl. Ed.).

[5] Stephenson D., Moon R.C., Teixeira A.F., Jawad M.A. (2014). Intussusception after roux-en-Y gastric bypass. Surg. Obes. Relat. Dis..

[6] Sohrabi C., Mathew G., Maria N., Kerwan A., Franchi T., Agha R.A. (2023). The SCARE 2023 guideline: updating consensus Surgical CAse REport (SCARE) guidelines. Int. J. Surg. Lond. Engl..

[7] Poliakin L.A., Sundaresan N., Hui B., IH McKillop, Thompson K., Gersin K. (2021 Aug). S146-Jejunojejunal intussusception after roux-En-Y gastric bypass: a case series of 34 patients. Surg. Endosc..

[8] Li X., Hu X., Fu C., Han L., Xie M., Ouyang S. (2023 Feb). Efficacy and safety of one anastomosis gastric bypass versus roux-en-Y gastric bypass for obesity: a meta-analysis and systematic review. Obes. Surg..

[9] Liagre A., Benois M., Queralto M., Boudrie H., Van Haverbeke O., Juglard G. (2022 Oct). Ten-year outcome of one-anastomosis gastric bypass with a biliopancreatic limb of 150 cm versus roux-en-Y gastric bypass: a single-institution series of 940 patients. Surg. Obes. Relat. Dis..

